# α-Ketoglutarate for Preventing and Managing Intestinal Epithelial Dysfunction

**DOI:** 10.1016/j.advnut.2024.100200

**Published:** 2024-03-02

**Authors:** Alejandro Bravo Iniguez, Min Du, Mei-Jun Zhu

**Affiliations:** 1 School of Food Science, Washington State University, Pullman, WA, United States; 2 Department of Animal Sciences, Washington State University, Pullman, WA, United States

**Keywords:** α-ketoglutarate, intestinal health, inflammatory bowel disease, colorectal cancer, gut microbiome

## Abstract

The epithelium lining the intestinal tract serves a multifaceted role. It plays a crucial role in nutrient absorption and immune regulation and also acts as a protective barrier, separating underlying tissues from the gut lumen content. Disruptions in the delicate balance of the gut epithelium trigger inflammatory responses, aggravate conditions such as inflammatory bowel disease, and potentially lead to more severe complications such as colorectal cancer. Maintaining intestinal epithelial homeostasis is vital for overall health, and there is growing interest in identifying nutraceuticals that can strengthen the intestinal epithelium. α-Ketoglutarate, a metabolite of the tricarboxylic acid cycle, displays a variety of bioactive effects, including functioning as an antioxidant, a necessary cofactor for epigenetic modification, and exerting anti-inflammatory effects. This article presents a comprehensive overview of studies investigating the potential of α-ketoglutarate supplementation in preventing dysfunction of the intestinal epithelium.

**Statement of Significance**

This article summarizes research findings on the health-beneficial effects of α-ketoglutarate in maintaining intestinal homeostasis and explores the associated underlying mechanisms.

## Introduction

The intestinal epithelium mediates the absorption of nutrients while serving as a barrier against foreign, potentially harmful compounds present in the lumen [1]. Proper intestinal barrier function is vital for overall health and the prevention of chronic inflammatory responses [2]. Unfortunately, the prevalence of gut barrier dysfunction continues to rise [3]. The intestinal epithelium is affected by multiple factors, including dietary, immunological, and microbial elements [2]. This underscores the need for intervention strategies that protect the intestinal epithelium against impairment and promote its recovery to normal conditions following exposure to stressors.

In recent years, the health-beneficial effects of α-ketoglutarate (AKG), an intermediate in the tricarboxylic acid (TCA) cycle, have garnered increasing recognition. These beneficial properties include the attenuation of adipose tissue weight gain in high-fat diet-fed mice [4,5], the extension of lifespans in flies [6], and the inhibition of angiogenesis in cancer cells through the modulation of hypoxia-inducible factor 1-α and vascular endothelial growth factor expression under hypoxia conditions [7]. Along with these effects, AKG displays both antioxidative and anti-inflammatory properties [[8], [9], [10]]. This article aims to provide a comprehensive overview of existing studies that explore the beneficial effects of AKG on intestinal health. It will shed light on the mechanisms through which AKG maintains epithelial homeostasis and combats various disease conditions.

### AKG chemistry

AKG, an intermediate of the TCA cycle, exerts a variety of effects [11]. It aids in the production of amino acids, acts as an antioxidant agent, and regulates gene expression related to development and aging [12]. Additionally, AKG functions as a bridge, linking carbon metabolism with nitrogen metabolism [13]. In the intestines, AKG serves diverse functions, including promoting protein synthesis and acting as an oxidative fuel source [14].

**FIGURE 1:**
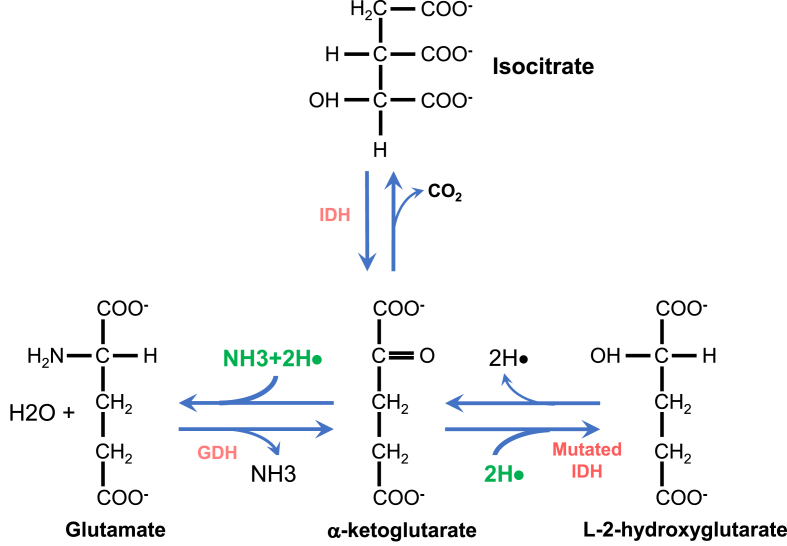
Synthetic pathways of α-ketoglutarate. Isocitrate dehydrogenase (IDH) catalyzes the reversible oxidative decarboxylation of isocitrate. Glutamate dehydrogenase (GDH) catalyzes the reversible oxidative deamination of glutamate.

The synthesis of AKG primarily occurs through 2 pathways: oxidative decarboxylation of isocitrate or oxidative deamination of glutamate (Figure 1). Isocitrate dehydrogenase (IDH) catalyzes the former, whereas glutamate dehydrogenase mediates the latter [15,16]. The intestinal epithelium receives additional sources of AKG from gut bacteria synthesis and dietary intake [17,18]. α-Ketoglutaric acid, the conjugate acid of AKG, is present at low yet detectable concentrations in many fruits and vegetables, although in meager amounts compared to other organic acids [18]. Given its involvement in the TCA cycle, AKG is a common metabolite, but levels decline with age [19].

Studies have begun exploring the potential of AKG as a supplement. Unlike glutamine, which displays poor stability in water, AKG is stable and soluble [20]. Upon ingestion, dietary AKG is largely absorbed and metabolized in the small intestine, with low concentrations entering the bloodstream [21]. In human studies, pure AKG or AKG salts are used at doses ≤30 g/d [[22], [23], [24]].

### The intestinal epithelium in healthy and diseased states

The intestinal epithelium plays a vital role in facilitating nutrient absorption while serving as a protective barrier against foreign potentially harmful compounds present in the lumen [1]. Both physical and chemical barriers, maintained by the epithelial cells of the intestines, segregate the underlying host tissue and immune system from contact with exogenous agents capable of compromising homeostasis [25]. Physical barriers include tight junction proteins linking epithelial cells together and mucus produced by goblet cells [26,27], and chemical barriers are established by Paneth cells that secrete antimicrobial peptides [28].

In most mammals, the intestinal epithelial cells have a relatively short lifespan, typically lasting 2–6 d before undergoing shedding. These cells are constantly renewed through the proliferation of stem cells located in the crypts throughout the intestinal tract [29,30]. Both the small and large intestines contain crypts, but villi, finger-like protrusions that extend out into the intestinal lumen, are exclusive to the small intestine. The crypt-residing stem cells give rise to various differentiated cell types, each with specialized functions. Among these, goblet cells and Paneth cells, integral for protective functions, are part of the secretory cell lineage. The secretory lineage also includes hormone-producing endocrine cells [31]. The majority of differentiated intestinal epithelial cells are enterocytes, primarily responsible for the absorption of nutrients [31,32].

The cells of the intestinal epithelium directly interact with microbial communities and their metabolites, exerting influence over host immune responses. For example, exposure to pathogens such as *Listeria monocytogenes* and *Salmonella* causes the secretion of IL-8, promoting leukocyte infiltration [33]. Bacterial LPS binds to receptors on intestinal epithelial cells, stimulating a proinflammatory signaling cascade involving the activation of nuclear factor kappa-light-chain-enhancer of activated b [34]. Along with the production of chemokines and cytokines, contact with bacteria triggers the production of reactive oxygen species (ROS), which further stimulate signaling cascades such as those involved with damage repair [35]. As an important mediator between the gut lumen and the host, the intestinal epithelium is critical for overall health, and its impairment characterizes different disease states.

Many diseases, including autoimmune disorders such as inflammatory bowel diseases (IBDs) and diabetes, impair gut epithelial barrier function [[36], [37], [38]]. IBDs encompass 2 conditions, Crohn’s Disease (CD) and Ulcerative Colitis (UC), which are characterized by specific components of the gastrointestinal tract they affect [39]. CD may intermittently manifest across the various components of the gastrointestinal tract, whereas UC is confined to the colon. CD often involves ulceration and severe bleeding, whereas UC is associated with complications like abscesses and fistulas [39,40]. Although the exact etiologies of IBDs remain to be fully elucidated, a combination of genetic, environmental, and lifestyle factors contributes to the dysregulation in innate and adaptive immune responses, driving these conditions [41]. Effective management is vital to mitigate the risk of further complications. Severe IBD increases the risk of overall colonic cancer development, and the roles of both chronic inflammation and immunosuppressors behind the development of cancers such as colorectal cancer (CRC) are being investigated [42,43].

CRCs pose a serious threat to a large proportion of the global population. In 2018, CRCs ranked as the second leading cause of cancer-related death [44]. Although early screening and the promotion of lifestyle changes have proven effective in reducing CRC incidence in some developed countries, others have witnessed an increase in cases of early-onset CRCs [45]. Risk factors contributing to the development of CRCs include personal and family medical history, dietary patterns, cigarette use, alcohol consumption, physical inactivity, as well as socioeconomic factors [46].

CRC is considered a heterogeneous disorder characterized by diverse causative mechanisms leading to its development. Genetic and epigenetic approaches have identified distinct subsets within the disease [47]. The initiation of most CRCs involves the formation of polyps, and the removal of these polyps, known as polypectomy, has proven to be an effective strategy in reducing CRC mortality [46,48]. Multiple genes have been identified as contributors to the initiation and progression of CRC, with adenomatous polyposis coli (APC) mutations being commonly found in a vast majority of CRC cases [49]. Proto-oncogene B-Raf mutations and the methylation of CpG islands at gene promoters are frequently detected in polyps and tumors [50]. Aberrant Wnt/β-catenin signaling contributes to metabolic reprogramming favoring glycolysis, a phenomenon known as the Warburg effect found in CRCs, partially through upregulation of pyruvate dehydrogenase kinase 1 [51]. Mutations in IDH1 are also observed in cancers, leading to elevated levels of the oncometabolite, R(−)-2-hydroxyglutarate (2HG) [52]. The 2HG, a competitive inhibitor of AKG, promotes colorectal tumorigenesis [53]. The frequency of specific mutations and epigenetic modifications differ across various demographics [50]. In addition, patients with CRC experience alterations in the intestinal environment, including changes in microbial and metabolite profiles [54]. The intricate and diverse mechanisms by which CRCs can develop highlight the importance of safeguarding the integrity of the intestinal epithelium against dysfunction.

### Effects of AKG on mediators of inflammation

Proper protection against harmful agents present in the lumen, such as free radicals or microbial compounds, requires a controlled immune response. Improper immune responses can result in unchecked inflammation, a characteristic feature of conditions such as CD and UC. The immune homeostasis of the gut involves communication between epithelial cells and immune cells, including T cells and macrophages. These cells dictate immune responses and impact each other largely through the production of cytokines [55]. The dysregulation of these cytokines can contribute to the inflammation characteristic of CD and UC. CD patients display elevated concentrations of T helper (Th)1 and Th17 cytokines, whereas UC patients tend to exhibit increased concentrations of Th2 cytokines [56]. CD tissue displays elevated concentrations of proinflammatory IL-17, IL-23, and TNFα, whereas UC tissue shows elevated proinflammatory IL-4 and IL-13 [56,57].

Macrophages are ubiquitous immune cells found in almost all tissues, performing both immune phagocytic activity and tissue-specialized function [58,59]. Through the production and release of cytokines and other signaling molecules, they regulate immune responses such as inflammation as well as the proliferation of epithelial progenitors [60]. Macrophages can polarize into either proinflammatory M1 macrophages or anti-inflammatory M2 macrophages depending on the stimuli [61]. Apart from opposite effects on inflammatory status, M1 and M2 macrophages exhibit metabolic differences. M1 macrophages favor glycolysis, whereas M2 macrophages demonstrate greater mitochondrial oxidative phosphorylation and fatty acid oxidation [62]. Dysregulated levels and activity of M1 macrophages can impair barrier function and contribute to intestinal diseases, making them potential targets for the management of inflammation and leaky gut [63].

Dietary AKG is effective in controlling the proinflammatory signaling molecules in different animal models. Aged female mice receiving a 2.0% AKG diet exhibited longer lifespans paired with a reduced serum proinflammatory cytokine profile compared to controls [64]. In dextran sulfate sodium (DSS)-induced colitis mice, both 0.5% and 1.0% AKG added to their drinking water ameliorated the induction of proinflammatory IL-1α, IL-6, IL-17A, and IL-18 [65,66]. DSS-treated mice provided with 1.0% AKG loss less weight than their counterparts [66]. *Citrobacter rodentium*-induced colitis mice receiving 0.5% AKG in their drinking water ameliorated colon shortening and tight junction protein suppression [67]. TNFα, interferon-γ, and proinflammatory IL concentrations in serum were also decreased [67]. In DSS/azoxymethane mice, 1.0% AKG supplementation reduced inflammatory index and tumor number, associated with decreased concentrations of IL-6, IL-22, IL-1b, and TNFα [68]. In piglets, the LPS challenge increased phosphorylation of nuclear factor kappa-light-chain-enhancer of activated B p65, which was not observed in challenged pigs supplemented with 1.0% AKG [10]. LPS challenge also decreased ileal villus height while upregulating concentrations of proinflammatory cytokines such as IL-6 and TNFα. Alterations to villus height and cytokine profile were mitigated by the addition of 1.0% AKG to the diet [69]. Carp fed low-protein diets supplemented with 0.4% AKG exhibited reduced concentrations of IL-1b and IL-6α compared to carp on low-protein diets without AKG supplementation [70]. The addition of AKG to a low protein feed improved the growth parameters and body length [70].

Along with downregulating proinflammatory signaling molecules, AKG promotes anti-inflammatory cytokine concentrations. IL-10 is an important anti-inflammatory cytokine that suppresses the transcription of proinflammatory genes, and its knockout in mice results in the spontaneous development of intestinal inflammation resembling CD [71,72]. AKG supplementation restored intestinal IL-10 concentrations in piglets challenged with LPS [69]. In LPS-challenged piglets, dietary AKG promoted regulatory T cell differentiation and suppressed Th17 cell differentiation in the intestine [69]. Aged mice receiving AKG showed higher concentrations of IL-10 secreted by splenic T cells [64]. Moreover, treatment with AKG upregulated IL-10 in both cerebral and hepatic tissues subjected to ischemia/reperfusion [73,74]. This effect was further confirmed in human neuroblastoma SH-SY5Y cells, where transfection with IL-10 small interfering RNA significantly weakened AKG’s ability to mitigate inflammation and apoptosis caused by Oxygen-Glucose Deprivation/Reoxygenation, highlighting the mediating roles of IL-10 [74].

AKG supplementation suppresses the M1 polarization elicited by bacteria and bacterial LPS [75,76]. In adipocytes, AKG promotes M2 polarization and upregulates concentrations of ten-eleven translocation (TET) enzymes [77]. These AKG-induced alterations to macrophages involve the promotion of fatty acid oxidation and epigenetic modifications [75,78]. In DSS-challenged mice, supplementation with 1.0% AKG reduced markers of M1 macrophages and increased markers of M2 macrophages in colon tissues [66]. Additionally, staining of colonic tissue for the membrane protein F4/80, a pan macrophage marker, revealed lower macrophage infiltration in the mice that received AKG [66]. Similarly, supplementation with 0.5% AKG ameliorated macrophage infiltration in the colon of mice challenged with *C. rodentium,* as demonstrated by reduced F4/80^+^ and CD11b^+^ staining [67]. Furthermore, 0.5% AKG in drinking water decreased concentrations of CD11b^+^CD64b^+^ macrophages in the spleen of mice subjected to DSS-induced colitis [65]. CD64 serves as a marker for proinflammatory M1 macrophages [79].

**FIGURE 2:**
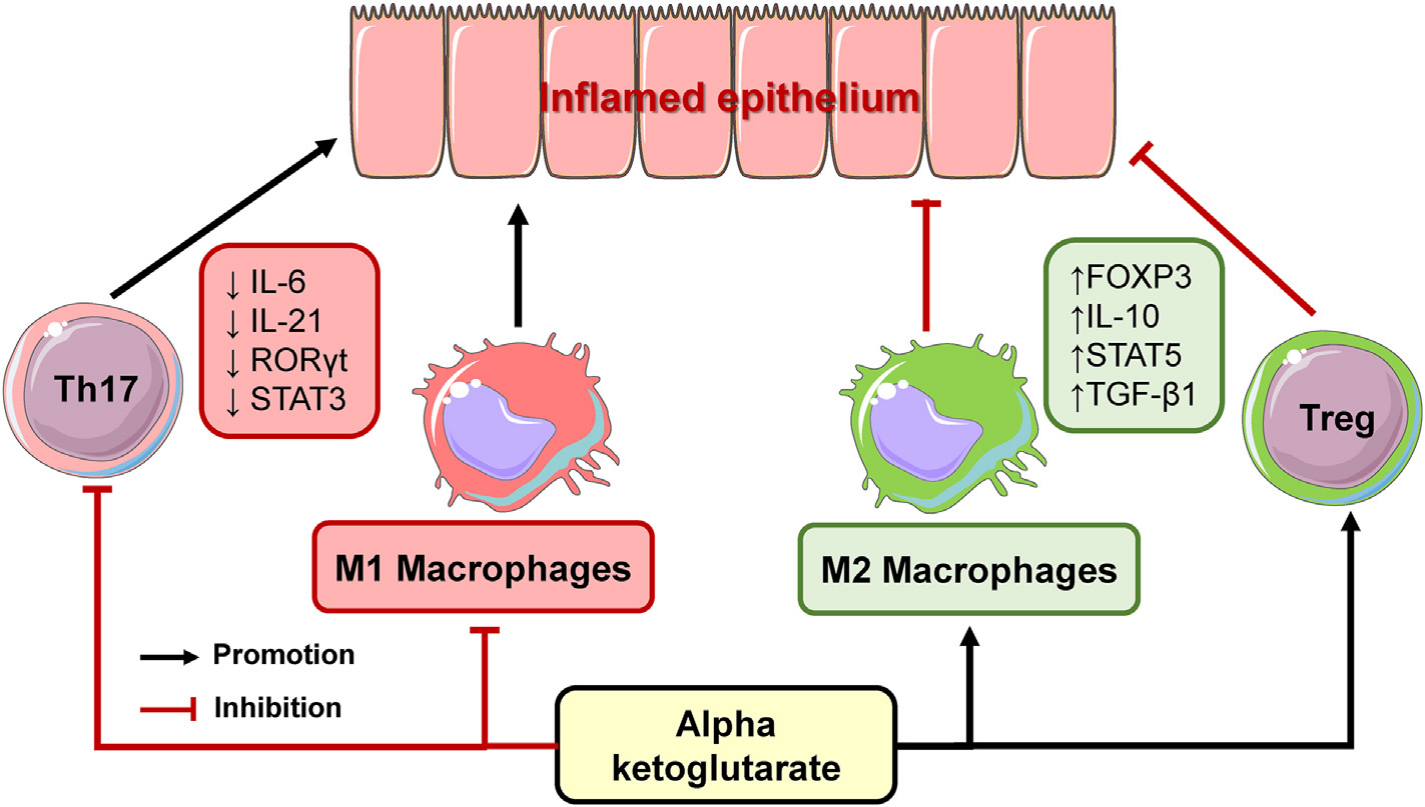
Potential mechanisms by which α-ketoglutarate (AKG) ameliorates intestinal inflammation through targeting macrophage polarization and T cell differentiation. AKG promotes polarization into anti-inflammatory M2 macrophages through epigenetic modifications and metabolic reprogramming. It also shifts the balance between T cells 17 (Th17) and regulatory T cells (Treg) by downregulating IL-6 and RORγt while upregulating TGF-β1/STAT5/FOXP3 signaling. These AKG-induced changes elevate concentrations of anti-inflammatory cytokines such as IL-10 and lower concentrations of proinflammatory cytokines such as IL-17. Black arrows represent promotive effects. Red lines represent inhibitory effects. FOXP, forkhead box P; IL, interleukin; RORγt: RAR-related orphan recetor γ; STAT5: signal transducer and activator of transcription 5; TGF-β, transforming growth factor-β.

In summary, AKG targets multiple components of the immune system, such as T cells and macrophages, to combat dysregulated responses and minimize the production of proinflammatory signaling molecules (Figure 2). Through these mechanisms, AKG mitigates damage to the intestinal epithelium in various animal models of intestinal inflammation. Despite its demonstrated ability to combat damage induced by different stressors in mice, pigs, and carp, the potential of AKG to manage symptoms in patients with IBD remains largely unexamined.

### Antioxidant effects of AKG

Excessive reactive molecules such as ROS lead to oxidative stress. Heightened oxidative stress and depletion of antioxidant concentrations are observed in various disease states, including IBDs [80,81]. ROS exposure can cause DNA damage [82]. Macrophages produce ROS to kill bacteria [83]. Mitochondria also are a major source of free radicals because of electron leakage during oxidative phosphorylation [84,85]. Under normal conditions, mitochondrial antioxidant defenses elicited through transcription factors such as nuclear factor erythroid 2-related factor 2 (NRF2) act as a safeguard against excessive oxidative stress [86]. NRF2 is an important transcription factor that, when stabilized, coordinates the induction of various antioxidant enzymes [87].

Proliferating cells located in crypts predominantly rely on glycolysis for their energy needs. As cells transition toward the apex of the villi, there is a shift toward oxidative phosphorylation, mirroring the gradient of cell proliferation compared with differentiation [88]. The transcriptional coactivator peroxisome proliferator-activated receptor-γ coactivator 1-α regulates mitochondrial biogenesis and exhibits a similar pattern of expression, with low levels in crypts and high levels in villi [89]. The high energy demand of the epithelium’s renewal and the presence of foreign bacteria in the lumen pose a risk for the overproduction of free radicals. Thus, antioxidant defense is crucial to prevent dysfunction.

AKG possesses the ability to directly scavenge free radicals and reduce oxidized compounds [8]. It reacts directly with hydrogen peroxide to produce succinate and water [90,91]. Additionally, AKG offers protection against the formation of lipid peroxidation products in the liver and kidneys as measured by thiobarbituric acid reactive substances assay [9].

AKG demonstrates the ability to alleviate oxidative stress in different animal models, leading to the restoration of vital antioxidant enzymes (Table 1) [65, 67, 70, 92, 94]. For instance, in a mouse model of *C. rodentium*-induced colitis, the provision of 0.5% AKG in drinking water reduced serum concentrations of malondialdehyde and hydrogen peroxide [67]. Exposure to *C. rodentium* decreased mRNA amounts of glutathione synthetase, *Nrf2*, and its downstream target, NAD(P)H dehydrogenase quinone 1, in the colon, all of which were restored by AKG [67]. Improvements in goblet cell numbers and mucin production accompanied the improvements in antioxidant signaling conferred by AKG treatment [67]. Likewise, AKG protects against oxidative stress induced by DSS treatment, normalizing mRNA amounts of glutathione synthetase and NAD(P)H dehydrogenase quinone 1 [65]. Shortening of the colon and damage to the colonic epithelium caused by DSS treatment was also abrogated by dietary AKG [65]. In weaned piglets, the addition of 1.0 % AKG to the diet mitigated the negative effects of oxidative stress induced by hydrogen peroxide injection, mitigating the decline of serum concentrations of catalase and superoxide dismutase [92]. In piglets challenged with LPS, a diet supplemented with 1.0% AKG blunted damage to the liver and increased glutathione peroxidase activity in the liver [94]. In carp, a low-protein diet reduced antioxidant capabilities, including total superoxide dismutase, catalase, and glutathione peroxidase. The addition of 0.4% AKG restored antioxidant enzyme content and increased intestinal *Nrf2* mRNA amounts [70]. In vitro studies have also shown that AKG normalizes antioxidant activity and reduces ROS production in intestinal porcine epithelial cells exposed to hydrogen peroxide [92,95].

**TABLE 1:** In vivo studies demonstrating the antioxidant effects of dietary α-ketoglutarate

Model	Dose/delivery	Effect(s) of AKG supplementation	Citation
Piglets challenged with H_2_O_2_	1% in diet	Decreased serum MDA Increased serum T-AOC, CAT, and SOD	[92]
Mice challenged with DSS	0.5% in drinking water	Decreased serum MDA and H_2_O_2_. Increased colon GSH. Increased colon *GSS* and *Nqo1*	[65]
Mice challenged with *C. rodentium*	0.5% in drinking water	Decreased serum MDA and H_2_O_2_. Increased colon *GSS*, *Nrf2*, and *Nqo1*	[67]
Piglets challenged with LPS	1.0% in diet	Increased liver GSH-Px	[94]
Carp on a low-protein diet	0.4, 0.8, or 1.2% in diet	Increased intestinal SOD and GSH-Px	[70]

Abbreviations: AKG, α-ketoglutarate; *C. rodentium, Citrobacter rodentium;* CAT, catalase; DSS, dextran sodium sulfate; GSH, glutathione; GSH-Px, glutathione peroxidase; GSS, glutathione synthetase; H_2_O_2_, hydrogen peroxide; MDA, malondialdehyde; Nqo1, NAD(P)H Quinone dehydrogenase 1; *Nrf2*, nuclear factor (erythroid-derived 2)-like 2; SOD, superoxide dismutase; T-AOC, total antioxidant capacity.

Intervention with AKG contributes to the restoration of proper mitochondrial function. In vitro, 2 mM AKG restores mitochondrial function in porcine intestinal epithelial cells challenged with 100 *μ*M hydrogen peroxide, leading to the recovery of adenosine triphosphate (ATP) production and improvement in mitochondrial membrane potential [95]. Moreover, piglets challenged with LPS exhibited reduced intestinal ATP concentrations, which were partially restored by supplementation with 1.0% AKG [96]. In intestinal porcine epithelial cells, 2 mM AKG treatment suppressed the negative effects on respiration and ATP production induced by 200 *μ*M hydrogen peroxide exposure [92].

Unchecked mitochondrial dysfunction and the associated oxidative stress render the intestinal epithelium for further complications. As a radical-scavenging molecule and inducer of antioxidant pathways, AKG helps control levels of free radicals in biological systems. Through its ability to combat oxidative stress, AKG protects the intestinal epithelium from escalating damage.

### Modulation of microbiome and microbial metabolites by AKG

IBD patients, including those in remission, display reduced microbiome diversity, referred to as “dysbiosis” [97]. Individuals with IBDs exhibit alterations such as reduced concentrations of *Bifidobacterium*, whose species ferment carbohydrates and are largely regarded as probiotics [[98], [99], [100]]. Belonging to the phylum *Actinobacteria*, *Bifidobacterium* is a gram-positive bacterium that establishes itself in the human gut very early in life, exhibiting a negative association with low-grade inflammation [101].

Accompanying dysbiosis in individuals with IBD, the concentrations of some amino acids are elevated, whereas short-chain fatty acids (SCFAs), butyrate, and propionate are decreased [102]. Derived from microbial fermentation in the gut, SCFAs serve as vital energy sources for intestinal epithelial cells and play a crucial role in intestinal homeostasis [103,104]. Most notably, butyrate exerts different positive effects on the intestinal epithelium, such as promoting barrier function through the upregulation of Adenosine monophosphate-activated protein kinase (AMPK) phosphorylation, increasing mismatch repair proteins by inducing epigenetic modifications at gene promoters and promoting epithelial differentiation [[105], [106], [107]]. In recent years, targeting the gut microbiome through prebiotics and probiotics has been explored as a strategy for treating IBD [108].

Studies using animal models of disease and stress have reported modulation of the gut microbiome following AKG supplementation. In mice, exposure to DSS in drinking water not only induced colitis but also dysbiosis, characterized by increased *Turicibacter* and decreased *Lactobacillus* in colon content [65]. The intervention with different probiotic *Lactobacillus* strains ameliorates inflammation and damage in DSS-induced colitis mice [109,110]. The inclusion of 0.5% AKG in drinking water blunted the changes in *Lactobacillus* and *Turicibacter* abundance in mice exposed to DSS, potentially contributing to its ability to mitigate disease symptoms [65]. In a mouse model of experimental CRC, a diet supplemented with 1.0% AKG not only mitigated intestinal inflammation and tumor development but also promoted microbial diversity and richness [68]. Analysis of the extracted colon revealed that AKG increased the abundance of SCFA-producing bacteria and decreased concentrations of *Firmicutes*, *Escherichia*, and *Enterococcus* [68]. In unchallenged mice, the provision of 1.0 % AKG in drinking water decreased the fecal ratio of *Firmicutes* to *Bacteroidetes* [111]. The addition of AKG to drinking water also decreased weight gain, an effect that was abolished when antibiotics were introduced to simulate germ-free conditions [111].

In contrast, in pigs, a diet supplemented with 1.0% AKG in combination with allicin resulted in higher abundance of *Firmicutes* and reduced *Bacteroidetes* in cecum content [112]. Growing pigs on 1.0% AKG diets had increased abundance of *Lactobacillus* in both their cecum and ileum, with the former organ also exhibiting greater *Bifidobacterium* and reduced *Escherichia coli*, whereas the latter had higher *Firmicutes* [113]. The cecum content of pigs fed low-protein diets with the addition of AKG had increased *Bifidobacterium* and decreased *Escherichia coli* compared to the low-protein group [114]. In carp fed low-protein diets, a promotive effect of AKG on *Firmicutes* in intestinal content was reported [70]. The ratio of *Firmicutes* to *Bacteroidetes* is associated with gut homeostasis, with a lowered ratio commonly found in IBD [115]. Additional studies are needed to uncover the impact of AKG on the gut microbiome, especially the potential effects on the gut microbiome of humans.

Studies have also investigated the impact of AKG on the intestinal metabolome, particularly on SCFAs. When combined with allicin, dietary AKG supplementation in pigs increased cecal concentrations of butyrate and total volatile fatty acids compared to controls on a basal diet [112]. AKG supplementation in pigs on a low-protein diet increased cecal concentrations of valerate and isovalerate while concurrently reducing ammonia concentrations [114]. Similar results were also observed in growing pigs, with AKG supplementation leading to elevated cecal concentrations of butyrate and valerate alongside decreased ammonia [113]. Growing pigs fed AKG also exhibited increased butyrate concentrations and reduced ammonia concentrations in their ileum [113]. Urea, a natural byproduct produced by the body to eliminate harmful ammonia, can also adversely affect the integrity of the intestinal barrier [116]. Experimental colitis mice provided with AKG in their drinking water exhibited reduced concentrations of urea in their cecal content [117].

In recent years, there has been a surge in animal studies investigating the impact of dietary AKG on the gut microbiome (Table 2) [65, 68, 70, 111, 112, 113, 114], including its effects on the metabolome. Compelling evidence suggests that AKG increases the concentrations of SCFAs and the relative abundance of bacteria responsible for their production. However, the effects of AKG on the major phyla, *Firmicutes* and *Bacteroidetes*, are less certain. Inhibitory effects on *Firmicutes* concentrations were observed in mice given AKG [68,111], whereas pigs and carps receiving AKG displayed heightened concentrations [70,112,113]. The disparity in *Firmicutes* concentrations may stem from differences in the samples used for analysis. The mice studies analyzed the microbiome extracted from the colon and feces [68,111], whereas the pig and carp studies utilized content from the small intestine and cecum [70,112,113]. These findings underscore the need for further exploration into the interply between the microbiome and AKG.

**TABLE 2:** Impact of dietary α-ketoglutarate on microbial communities

Organism	Dose/delivery	Microbial changes	Citation
Carp	0.4% in diet	↑: (int con) *Aeromonas*, *Firmicutes*, *Actinobacteria*	[70]
		↓: (int con) *Citrobacter*, *Cetobacterium*, *Pseudomonas*, *Proteobacteria*	
Pigs	1% in diet	↑: (cec con) *Bifidobacterium*, *Lactobacillus*, (ile con) *Lactobacillus*, *Firmicutes*	[113]
		↓: (ile con) *Escheria coli*	
	1% in diet	↑: (cec con) *Bifidobacterium*	[114]
		↓: (cec con) *Escheria coli*	
	1% in the diet in combination with 0.5% allicin	↑: (cec con) *Firmicutes*	[112]
		↓: (cec con) *Bacteroidetes*	
Mice	1% in water	↑: (fec) *Bacteroidetes*	[111]
		↓: (fec) *Firmicutes*	
	1% in diet	↑: (col) *Verrucomicrobia*, *Actinobacteria*, *Akkermansia*, *Butyricicoccus*, *Clostridium*, *Ruminococcus*	[68]
		↓: (col) *Firmicutes*, *Escherichia*, *Enterococcus*	
	0.5% in drinking water before and throughout the DSS challenge	↑: (fec) *Lactobacillus*	[65]
		↓: (fec) *Turcibacter*	

Parentheses denote the source of microbial extraction.

Abbreviations: cec: cecum; col: colon; con: content; DSS, dextran sodium sulfate; fec: feces; ile: ileum; int: intestine; ↑: promotion; ↓: reduction.

### Beneficial effects of AKG on tight junction assembly

Tight junction proteins play a crucial role at the apical surface, serving as a barrier to seal cells together, establish polarity, and prevent leakage [118]. The tight junctions are primarily composed of 3 categories of proteins, including occludins, claudins, and junctional adhesion molecules. The expression of tight junction proteins is mediated by multiple signaling pathways and intracellular molecules, including those found in the lumen [119]. The assembly of tight junctions is hindered by proinflammatory cytokines such as TNFα [120]. The disruption to tight junctions and barrier function induced by proinflammatory cytokines involves increased phosphorylation of myosin II regulatory light chain (MLC) and upregulation of MLC kinase (MLCK) [121,122]. Both UC and CD patients exhibit increased MLCK expression and activity in their affected epithelial tissue [123], accompanied by reduced expression of tight junction proteins such as junctional adhesion molecule A [124]. Phosphorylation of MLC jeopardizes epithelial barrier function through tight junction remodeling, leading to increased permeability [125]. To control inflammation and safeguard barrier function, cells produce anti-inflammatory cytokines. Anti-inflammatory IL-10 downregulates proinflammatory genes through Janus Kinase 1/signal transducer and activator of transcription 3 signaling [126,127]. The importance of IL-10 is further exhibited by IL-10 deficient mice, which spontaneously develop colitis and exhibit lower RNA and protein levels of tight junction proteins [128,129].

**FIGURE 3:**
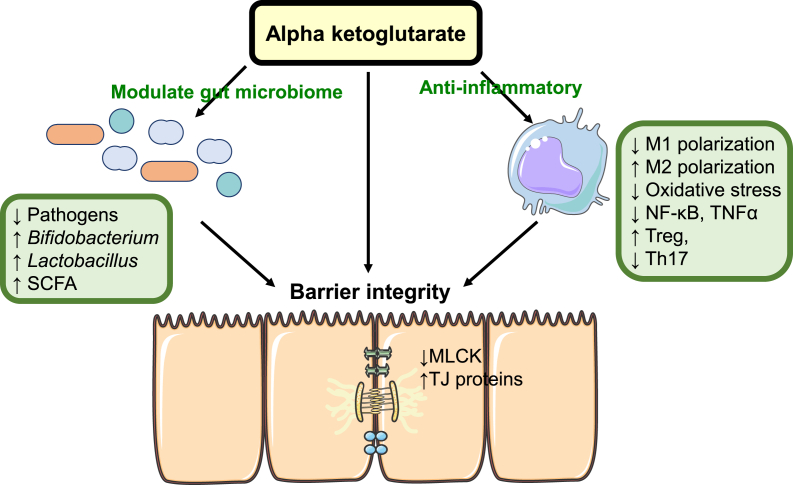
Hypothesized mechanisms underlying the enhancement of intestinal epithelial function by α-ketoglutarate (AKG). AKG safeguards epithelial barrier function through both direct and indirect mechanisms. It reduces the activity of myosin light-chain kinase (MLCK) and enhances tight junction (TJ) protein concentrations. Additionally, AKG mitigates inflammation and modulates the microbiome, further supporting the intestinal epithelium, decreasing concentrations of stressors that hamper barrier function, and increasing levels of short-chain fatty acids (SCFAs), which function as energy substrates. NF-κB, nuclear factor kappa-light-chain-enhancer of activated B; TNFα, tumor necrosis factor α; Treg, regulatory T cell; Th17, T helper 17 cell.

In addition to its anti-inflammatory properties and regulation of gut microbiota, AKG enhances barrier function by modulating the expression of tight junction proteins (Figure 3). In a study involving female mice exposed to DSS to induce colitis, the addition of 1.0% AKG in drinking water upregulated colonic occludin and E-cadherin compared to challenged controls [66]. Similarly, male mice receiving 0.5% AKG in their drinking water exhibited a mitigated impairment of tight junction protein levels and protection against DSS-induced colitis [65]. In early-weaning pigs, a diet containing 1.0% AKG ameliorated LPS-induced reduction of occludin, claudin-1, claudin-3, and claudin-7 protein levels in the small intestine [130]. The protection of intestinal tight junction protein levels by AKG was also observed in the duodenum of rats undergoing gastric bypass surgery [131]. Furthermore, carp on 1.0% AKG diets, challenged with *Aeromonas hydrophila* infection, had improved expression of various tight junction proteins, including zonula occludens and claudin-1, compared to challenged carp on normal diets [93]. The addition of AKG in the diet also mitigated MLCK upregulation induced by infection with *A. hydrophila* [93]. Although the capacity of AKG to enhance tight junction protein hasn’t been explored as extensively as its anti-inflammatory or antioxidant properties, the existing literature reveals promising potential. In various models of intestinal disease, dietary AKG demonstrated the ability to mitigate the downregulation of tight junction proteins in the small and large intestines. However, our understanding of the underlying mechanisms is limited. The suppression of MLCK and subsequent phosphorylation of MLC may be a crucial factor contributing to the positive effects of AKG against leaky gut.

### Impact of AKG on pathways and mechanisms regulating proliferation and differentiation balance

An array of signaling pathways intricately regulates the delicate balance between proliferation and differentiation to maintain epithelial homeostasis. Among these pathways, canonical Wnt signaling plays a central role in modulating epithelial homeostasis by regulating the expression of multiple genes related to proliferation [1,132]. Wnt signaling is highest in the crypts, where progenitor stem cells reside and proliferate [133,134]. Transforming growth factor-β/bone morphogenic protein signaling serves as a counterbalance, downregulating Wnt/β-catenin signaling as cells migrate away from the base of the crypts [[135], [136], [137]]. Activation of the Wnt pathway stabilizes β-catenin, enabling its nuclear translocation and subsequent formation of a complex with the T cell factor/lymphoid enhancer factor family to initiate gene expression [138,139]. In the absence of Wnt signaling, β-catenin is targeted for degradation by a complex composed of APC, axin, casein kinase 1, and glycogen synthase kinase 3 [49,132]. Bone morphogenic protein signaling is also required for proper villus formation [140], and Wnt plays a critical role in the proper differentiation of Paneth cells [141].

As intestinal epithelial cells migrate away from the crypts, there is a concomitant decrease in Wnt signaling while differentiation signaling increases [134]. The transcription factor atonal homolog 1 (Atoh1) plays a central role in driving the differentiation of secretory lineage cells. Notch signaling, however, acts as a suppressor of Atoh1, favoring the differentiation of absorptive lineages at the expense of the secretory lineages [142]. Acting as a transmembrane receptor, Notch downregulates Atoh1 through activation of hairy enhancer of split 1, inhibiting targets such as Krüppel-like factor 4, a transcription factor required for goblet cell differentiation [143,144]. Key ligands for Notch include δ-like1 (DLL1), DLL4, and jagged1. The ablation of DLL1 augments intestinal goblet cell concentrations [145]. In contrast to other differentiated secretory cells, Paneth cells reside within the crypts and express both DLL4 and Wnt3, thereby contributing to the support of the intestinal stem cell niche [146]. In summary, the balance between proliferation and differentiation in intestinal epithelial cells is carefully orchestrated to maintain the intestinal epithelium.

AKG supplementation effectively countered abnormal stemness and activation of Wnt signaling in a mouse model of colitis [147]. It mitigated the induction of β-catenin signaling in DSS-induced colitis mice, and co-administration of the β-catenin agonist, SB-216763, nullified the beneficial effects of AKG against colon damage [65,66]. In *Apc*^*Min/+*^ mice, intraperitoneal injection of 400 mg/kg AKG reduced the development of intestinal tumors and downregulated Wnt signaling [147]. In intestinal organoids culture in vitro, AKG is beneficial against TNFα stimulation, including the downregulation of β-catenin [67]. In addition to targeting Wnt/β-catenin to ameliorate colitis, AKG treatment also modulates related metabolic signaling. The addition of 1.0% AKG to the drinking water of DSS-challenged mice downregulated mRNA amounts of pyruvate dehydrogenase kinase 1 and upregulated protein expression of IDH1 [66]. AKG additionally promoted a transition away from glycolysis and toward oxidative phosphorylation in the colon of challenged mice [66].

Effects of AKG on differentiation are less explored. In *C. rodentium-*induced colitis mice, the inclusion of 0.5% AKG in drinking water mitigated goblet cell dysfunction and restored mRNA amounts of Mucin 2 (*Muc2)* and *Muc3* [67]. Similar outcomes were observed in DSS-challenged mice, where 1.0% AKG in drinking water promoted the expression of *Muc2* as well as goblet cell markers, Krüppel-like factor 4, and Trefoil factor 3, in the colon [117]. In *Apc*^*Min/+*^ mice organoids, AKG treatment upregulated differentiation-related genes and decreased stemness [147]. AMPK, a regulator of metabolism, also exerts a positive influence on intestinal epithelial differentiation through the upregulation of transcription factors dictating differentiation [148]. In flies, AKG upregulates *AMPKα* and downstream targets [6]. In piglets, a 1.0% AKG diet ablated induction of AMPK phosphorylation by LPS in the small intestine [95]. Additional studies should continue to uncover the mechanisms by which AKG promotes secretory cell differentiation.

Preserving the balance of proliferation and differentiation is crucial for maintaining intestinal homeostasis. Perturbations to this balance are evident in intestinal disease states, often manifesting as excessive proliferation. AKG treatment downregulates Wnt/β-catenin and glycolysis signaling and favors differentiation. The upstream targets responsible for this shift remain to be elucidated.

### Epigenetic modifications induced by AKG

Epigenetic modifications, such as DNA methylation, are instrumental in regulating gene expression without causing permanent changes to the DNA sequence. Distinct methylation patterns distinguish genes in the biopsy tissue of IBD patients from controls [[149], [150]]. For example, children with UC exhibited elevated CpG methylation of *Muc2* in colonic epithelial cells compared to healthy controls [151]. AKG serves as a vital cofactor for enzymes such as TET1, TET2, and TET3, which play a pivotal role in catalyzing DNA demethylation [152]. These 3 TET enzymes are oxygenases responsible for converting 5-methylcytosine to 5-hydroxymethylcytosine (5hmC) [153,154]. In certain cancers, such as pancreatic ductal adenocarcinoma, diminishing concentrations of 5hmC parallel the progression from benign to malignant stages [155]. Mice lacking TET2 exhibited exacerbated symptoms, increased inflammation, and higher IL-6 expression after exposure to DSS compared to wild-type counterparts [156]. The oncogenic metabolite, 2HG, inhibits TET enzymatic activity, resulting in decreased 5hmC [157].

A limited amount of studies have explored the impacts of AKG on epigenetic status. In cultured macrophages, AKG supplementation elevated TET protein concentrations and promoted demethylation [77]. In DSS-treated mice, the inclusion of 1.0% AKG in drinking water reduced colonic concentrations of 2HG [66]. In intestinal organoids, AKG treatment mitigated glutamine deprivation-induced stemness and hypermethylation [147]. Additionally, AKG treatment decreased DNA hypermethylation of Wnt antagonists, tumor suppressors genes, and genes related to differentiation, leading to increased expression compared to controls [147].

Epigenetics is a novel area of study that has gained increased focus in recent years. Because of its identification as a necessary cofactor for demethylation, studies have begun exploring how AKG treatment impacts the epigenetics of genes regulating different processes, such as aging, development, and differentiation [19]. The exploration of how AKG and its harmful, competitive inhibitor 2HG induce epigenetic changes driving intestinal homeostasis is still in its early stages, which warrants further research to establish a more comprehensive understanding.

In conclusion, ensuring proper intestinal epithelium function is paramount for overall health. The maintenance of intestinal epithelium homeostasis hinges on a delicate balance between cell proliferation and differentiation. Disturbances in these regulatory pathways manifest in various intestinal pathologies, contributing to dysfunction and setting the stage for further complications. Given the significant impact of conditions like IBDs and CRC on the gut, there is a pressing need for additional strategies to fortify the intestinal epithelium. In vitro experiments and animal models of intestinal disease suggest the potential of AKG to enhance gut epithelial health. AKG exerts immunomodulatory effects, downregulating proinflammatory cytokine production and shifting macrophage polarization away from the proinflammatory M1 state. It further safeguards the intestinal epithelium against damage by upregulating antioxidant pathways. AKG mitigates dysfunctional signaling, including aberrant proliferation and downregulation of tight junction proteins. In addition, AKG alters metabolic pathways, gene methylation status, and the microbiome, but additional investigation is warranted to fully understand how these contribute to the beneficial effects of AKG. Moving forward, future studies should aim to fill gaps surrounding the ability of AKG to strengthen the intestinal epithelium and to test how to translate these promising findings from animal models to clinical trials and human applications.

## Author contributions

The authors’ responsibilities were as follows – ABI and M-JZ: conceptualized the article, wrote the manuscript, and designed the figures; MD: contributed to the revision of the manuscript; and all authors: read and approved the final manuscript.

## Conflict of interest

The authors report no conflicts of interest.

## Funding

This work was financially supported by the Washington State University Agricultural Research Center Emerging Research Issues Competitive Grant, the USDA-National Institute of Food and Agriculture (2018-67017-27517), and the National Institute of Health (R01-HD067449).
